# Syphilis and Pseudo-Syphilis

**Published:** 1884-09

**Authors:** 


*Syphilis and Pseudo-Syphilis.* By ALFRED COOPER, F.R.C.S. Pp. 339. London: J. & A. CHURCHILL. 1884.

PROBABLY on no single disease have more monographs appeared than on Syphilis. Difficult and complicated as is the subject in theory as well as in practice, it is still one which readily lends itself to literary handling. It has an interesting history, which can be discussed with some show of learning by the help of a few quotations; there is a considerable want of definiteness about its actual symptoms and progress, which leaves convenient openings for literary disquisition; and, lastly, there is an absence of that necessity for laborious and minute investigations into its pathological anatomy, which are not relished by the mere scribbler. For these reasons we are inclined to look with some suspicion on such a work as that before us, and all the more so, perhaps, that it is from the pen of one who is attached to a special hospital. But a perusal of the work dispels our suspicions. It is a really able work, containing nothing strikingly novel, but well and clearly written, and fully up to date.

There is little to be noted in the work in the way of quotation or criticism. We are somewhat doubtful of the propriety of the term Pseudo-Syphilis for a disease altogether different and distinct from Syphilis. Shall we ever get one definite name which will satisfy all men for the soft venereal ulcer? Here are the author's views—the views of the great majority of the profession, it need scarcely be said—on the relations of syphilis and the soft sore:

"1. The disease known as Syphilis is due to the action of a specific virus, a pathological agent, separate and distinct from every other animal poison, and never originating *de novo*.

"2. The soft venereal ulcer (pseudo-syphilis) is due to the action of irritating secretions, which, so far from being identical with the poison of syphilis, are altogether different and distinct from it. Such an ulcer may, and frequently does, arise *de novo* from inoculation of the products of inflammation, and may then be transmitted from one individual to another.

"3. The virus of syphilis may be commingled with the secretions of a pseudo-syphilitic sore, or with secretions which by themselves would cause such a sore. In either case the resulting ulceration would possess double properties, evidences of which would sooner or later be exhibited."

The propositions thus clearly enunciated are argued out with great freshness and vigour. On the important subject of syphilis and vaccination, the views of the author, that pure vaccine not mingled with blood will not convey infection, may have to be modified by the recent unfortunate experience of Dr. Cory. Otherwise, the whole of this section is most praiseworthy.

The chapters on syphilitic affections of the various organs and systems are particularly good, especially so the chapter on Syphilitic Affections of the Nervous System. The chapter on Hereditary Syphilis also is worthy of special mention for its excellence.

The chapter on the Prevention of Syphilis presents the thing in a nutshell:—" A large majority of the young men of the upper and middle classes suffer in youth from some form of venereal disease, and among the lower classes syphilis is terribly common and often very severe. . . . The source of the virus is prostitution. . . . No human means are sufficient to abolish prostitution. . . . Not only prostitution, but syphilis as well, can be diminished by properly regulated official supervision." *Ergo*—the conclusion is obvious.

An eminently practical chapter on Treatment concludes what is, perhaps, the most noteworthy work on Syphilis which has appeared in recent years.

